# Dioxin levels in meat samples of selected free-living and farmed cervids

**DOI:** 10.2478/jvetres-2025-0037

**Published:** 2025-08-16

**Authors:** Małgorzata Warenik-Bany, Szczepan Mikołajczyk, Marek Pajurek, Sebastian Maszewski, Ewelina Bigoraj

**Affiliations:** Department of Food and Feed Chemical Research, National Veterinary Research Institute, 24-100 Puławy, Poland

**Keywords:** dioxins, game meat, persistent organic pollutants, risk assessment

## Abstract

**Introduction:**

The aim of the research was to determine the levels of dioxins and polychlorinated biphenyls (PCBs) in muscle samples of free-living and farmed cervids and to estimate the health risk to consumers of food originating from these animals.

**Material and Methods:**

The research material was collected from red deer (*Cervus elaphus* L.) (n = 22), roe deer (*Capreolus capreolus* L.) (n = 6) and fallow deer (*Dama dama*) (n = 6). The isotope dilution technique, supported by high resolution gas chromatography coupled with high resolution mass spectrometry was used.

**Results:**

The average concentration of polychlorinated dibenzo-p-dioxins, polychlorinated dibenzofurans and dioxin-like PCBs in the muscles of free-living cervids was 4.77 ± 2.92 pg World Health Organization toxic equivalency quotients (WHO-TEQ)/g fat, and in the muscles of farmed cervids was 1.85 ± 1.21 pg WHO-TEQ/g fat. Of the tested samples taken from free-living animals, approximately 13% did not meet the requirements of EC Regulation No. 2023/915 EU as they exceeded the maximum limit for congeners.

**Conclusion:**

Both frequent and occasional consumption of meat obtained from free-living and farmed cervids do not pose a threat to human health, because estimated intake of the analysed congeners is very low from these sources. However, very frequent consumption of highly contaminated free-ranging wild game muscles can pose a health risk (in this pattern, an adult consumes dioxins at 133% of the tolerable weekly intake (TWI) and a child at 202% of the TWI).

## Introduction

The development of civilisation has resulted in the release of chemical pollutants into the natural environment, including polychlorinated dibenzo-p-dioxins (PCDD), polychlorinated dibenzofurans (PCDF) and dioxin-like (dl-) and non-dioxin-like (ndl-) polychlorinated biphenyls (PCBs). Because of their physicochemical properties, they are resistant to chemical and biological transformations and spread very easily around the globe. Their lipophilicity causes them to bioaccumulate and biomagnify in the aquatic and terrestrial food chains. Dioxins and PCBs are resistant to the action of bacterial flora and digestive acids present in the digestive tract of animals. Transported in the blood along with lipoprotein particles, they are deposited in fat-rich tissues and in the liver (24). The concentration of these harmful substances in animal tissues is often higher than in the surrounding environment, which is why food from animals inhabiting areas polluted industrially or agriculturally may be a serious source of dioxins and PCBs for consumers (1, 2, 12, 23, 29).

Dioxins and PCBs are neurotoxic and carcinogenic and can cause many toxic effects, including disruption of the functions of the endocrine, immune, cardiovascular, skeletal, reproductive and central nervous systems, and the maldevelopment of various organs (1, 2, 23). In 2013 the European Food Safety Authority (EFSA) recognised dioxins as compounds with the highest potential threat to human health as regards chemical hazards present in the meat of bovine animals (10). In 2018 the tolerable weekly intake (TWI) was reduced sevenfold from 14 pg World Health Organization toxic equivalency (WHO-TEQ)/kg body weight (b.w.) per week to 2 pg WHO-TEQ/kg b.w. per week (12). This proves the need to limit consumers’ exposure to dioxins, as well as the need to identify the type of foods that may constitute a significant source of dioxins and PCBs for consumers.

A review of global and national literature on the presence of dioxins, polychlorinated dibenzofurans and polychlorinated biphenyls (PCBs) in food indicates that special attention should be paid to food products obtained from game animals (29). Meat or derivatives from game animals that feed in industrially or agriculturally polluted areas may be a major source of dioxins and PCBs for humans (8, 9, 11). In recent years, such meat has become an increasingly popular food for humans, which is why in 2023 the European Commission introduced maximum levels (limits) for food products from cervids, wild game birds and wild boars (*Sus scrofa*) (7).

Game meat is rich in easily digestible total protein, low in fat and contains three times as many micro- and macro elements than meat originating from intensively fed farm animals (4). Currently, the highest production of game meat takes place in Africa (about 53% of world production). Europe trails far behind, with production at about 7% of the world’s total. Poland is one of the leading producers and suppliers of meat obtained from the wild large mammals such as red, roe and fallow deer in Europe (25, 28). In the 2022/2023 hunting season, 7,821 tonnes of deer and roe deer meat were taken from prey in Poland (26). In addition to their hunting, game animals are also farmed. According to the Federation of European Deer Farmers Association, there are 200 large farms in Poland with 22,000 breeding animals, more than half (66%) being fallow deer (13). Despite Poland being one of the leading producers and exporters of game meat in Europe, the consumption of this type of meat in the country is low (0.08 kg/person per year) (4, 16, 20). The reasons for the negligible consumption of game meat are the lack of tradition in the consumption of this type of meat, the inability to prepare it, its high price and its narrow sales channel (4, 18). In addition, information about zoonoses and the presence of dioxins and PCBs disseminated by the media raises many concerns about the safety of consumption this type of food; therefore, the quality of game meat should be thoroughly tested in order to allay consumer concerns. With such an aim intended, the research sought to determine the levels of dioxins and PCBs in muscle samples of free-living and farmed cervids and to estimate the health risk to consumers of food originating from these animals.

## Material and Methods

**Fig 1.**
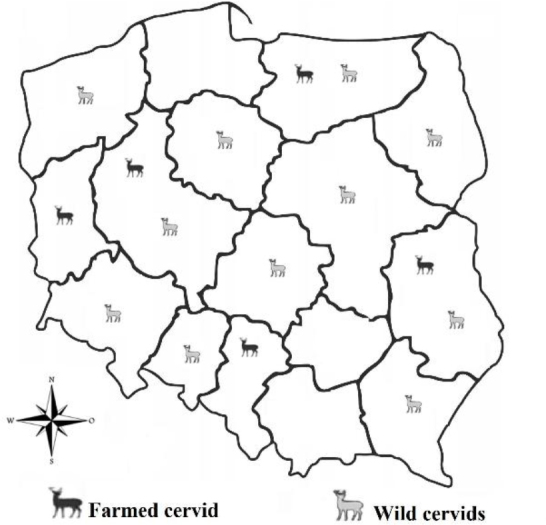
Sampling locations

The research material was collected from red deer (*Cervus elaphus* L.) (n = 22), roe deer (*Capreolus capreolus* L.) (n = 6) and fallow deer (*Dama dama*) (n = 6) from hunting districts and from deer farms located in all Polish voivodeships except Pomorskie, Świętokrzyskie and Małopolskie (Fig. 1). The animals were obtained during hunts as part of the annual plans of hunting clubs and as part of National Control Programme for Food of Animal Origin in the years 2010–2020. Samples were taken from 23 free-living and 11 farmed deer. The research material was approximately 200–300 g of muscle tissue, the samples of which were packed in polypropylene bags and frozen at a temperature of ≤–18°C.

### Reagents

All organic solvents and chromatographic sorbents used were of adequate purity for residue analysis. The reagents used were n-hexane, toluene, cyclohexane, dichloromethane, isooctane and methanol. Florisil was purchased from LGC Standards (Wesel, Germany). Carbopack C adsorbent and Celite 545 filter aid from Supelco (Bellefonte, PA, USA), silica gel from Fluka Feinchemikalien (Neu-Ulm, Germany) and diatomaceous earth from Restek (Bellefonte, PA, USA) were used. Concentrated 98% sulphuric acid and anhydrous sodium sulphate were procured from Merck (Darmstadt, Germany). The 99.9999% pure helium and 99.999% pure nitrogen used were from Messer (Gumpoldskirchen, Austria). Standards of PCDD/Fs and PCBs were obtained from the Cambridge Isotope Laboratory (Tewksbury, MA, USA) and Wellington Laboratories Inc. (Ontario, Canada).

### Analytes

There were seven analytes of interest: 2,3,7,8-substituted PCDDs, ten 2,3,7,8-substituted PCDFs, twelve dl-PCB congeners (PCB 77, 81, 105, 114, 118, 123, 126, 156, 157, 167, 169 and 189) and six ndl-PCBs (PCB 28, 52, 101, 138, 153 and 180).

### Analytical method

For the determination of PCDDs, PCDFs, dl-PCBs and ndl-PCBs, an accredited test method for detection and quantification, which was based on the isotope dilution technique with high resolution gas chromatography coupled with high resolution mass spectrometry (HRGC-HRMS) was used (Table 1). This method is considered the gold standard for the detection and quantification of PCDDs, PCDFs and dl-PCBs. The results of analytical tests for dioxins and dl-PCBs were presented in quantifiable units called World Health Organization toxic equivalency quotients (WHO-TEQ). The final content of dioxins and dl-PCBs is the sum of the concentrations of 29 toxic PCDD, PCDF and dl-PCB congeners multiplied by the toxic equivalency factor assigned to them (TEF_2005_) established by the World Health Organization. Non-dioxin-like-PCBs are expressed as the sum of the concentrations of six PCB congeners (28, 52, 101, 138, 153 and 180) (7). Muscle samples were minced and frozen at a temperature of ≤–18°C for 12 h, then freeze-dried for 48 h. The pressure during the freeze-drying process was maintained at 1 mbar. The temperature of the freeze dryer chamber was in the range of –40°C down to –60°C. After initial sample preparation, an appropriate sample weight was extracted using Accelerated Solvent Extractor (Dionex, Sunnyvale, CA, USA). The extraction mixture used was dichloromethane/hexane (50/50, v/v). To remove interferences, the obtained extracts were purified using multi-stage column chromatography. The purification process used silica gel modified with sulphuric acid, Florisil and Carbopack C. During purification on a Florisil column, the analytes were separated into two groups: PCDD/PCDFs and PCBs. In the next stage of purification on the Carbopack C/Florisil column, PCBs were further divided into fractions: non-ortho PCBs and mono-ortho PCBs. The extracts, purified and divided into three fractions, were concentrated after adding recovery standards and subjected to instrumental HRGC-HRMS analysis. The laboratory used a Trace Ultra GC gas chromatograph (Thermo Scientific, Milan, Italy) with an AS2000 or TriPlus autosampler (CTC Analytics, Zwingen, Switzerland) connected to a MAT95XP or DFS high-resolution mass spectrometer (Thermo Scientific, Bremen, Germany) with double focusing (magnetic and electric) and inverted Nier–Johnson geometry. The mass spectrometer was operated in electron ionisation mode under conditions providing a resolution exceeding 10,000 for the full range of mass spectra collected. The research method used meets the requirements of Commission Regulation (EU) 2017/644/(6).

**Table 1.** Selected parameters of the research method used

Validation characteristic		Validation results		
		PCDD/PCDFs	dl-PCBs	ndl-PCBs
Linearity		pg WHO-TEQ/g fat		ng/g fat
	Range	0.10–500.0	0.25–400.0	0.21–800.0
	Relative response rate	0.907–1.142	1.014–1.182	0.974–1.258
Limit of detection		pg/g		ng/g
		0.02	0.40	0.001
Limit of quantification		pg/g		ng/g
		0.05	1.00	0.003
Recovery (%)		60.0–120.0		60.0–120.0
Expanded uncertainty (k = 2) (%)		16.38	16.47	22.67

1 PCDD/PCDFs – polychlorinated dibenzo-p-dioxins and polychlorinated dibenzofurans; dl-PCBs – dioxin-like polychlorinated biphenyls; ndl-PCBs – non-dioxin-like polychlorinated biphenyls

### Quality control

Internal and external research quality control included evaluation of the recovery of internal standards labelled with the ^13^C carbon isotope (1,2,3,4-TCDD and PCB-111), analysis of blank reagent samples, use of certified reference materials (BCR-607 and T-0642; Institute for Reference Materials and Measurements, Geel, Belgium) and participation in proficiency tests organised by the EU Reference Laboratory for Persistent Organic Pollutants (POPs) in Feed and Food (Freiburg, Germany).

### Exposure assumptions

Exposure was estimated for adults (70 kg) and children (23.1 kg). Meat portions were 200 g for adults and 100 g for children. The frequency of consumption was taken into account and the consumers were divided into three groups: frequent (90 portions/year), periodic (12 portions/year) and occasional (2 portions/year) (29). To estimate exposure, the concentrations of the sum of PCDD/PCDF/dl-PCBs were used, calculated according to the upper-bound concept. The amount of each unquantified congener was its limit of quantification multiplied by its TEF. The average concentration value (X_avr_) and P97.5 of the total PCDD/PCDF/dl-PCB concentration were taken into account. For exposure assumptions, the results were converted to wet weight. Similarities in levels and profiles of contaminants in roe deer and fallow deer meat samples have been found, the results for these species were combined and exposure for wild and farmed cervids was calculated.

## Results

### Levels of dioxins and PCBs

The average concentrations of PCDDs, PCDFs and dl-PCBs expressed in accordance with EU regulations in pg WHO-TEQ/g of fat, and the average concentration of ndl-PCBs in ng/g of fat are presented in Table 2. The muscle tissue levels of the dioxins and furans measured together and of these measured together with dioxin-like biphenyls in free-living cervids were approximately threefold higher than the muscle tissue levels determined in farmed cervids. However, in the case of the non-dioxin-like biphenyls, the level was twice as high in free-living cervids as in farmed counterparts. The average concentrations of PCDD/PCDFs and PCDD/PCDF/dl-PCBs in the muscles of free-living cervids were 84% and 64%, respectively, of the maximum level set in Commission Regulation 2023/915/EU (7). The average concentrations of PCDD/PCDFs and PCDD/PCDF/dl-PCBs in farmed cervid muscles, however, were only 25% of the maximum level. Of the tested samples taken from free-living animals, approximately 13% did not meet the requirements of EC Regulation No. 2023/915 EU (7). After taking into account the method uncertainty, the determined concentrations of dioxins and the sum of dioxins and dl-PCBs exceeded the maximum limits for these pollutants.

**Table 2.** Levels of dioxins and polychlorinated biphenyls (PCBs) in the muscle tissue of free-living and farmed cervids

		PCDD/PCDFs	dl-PCBs	PCDD/PCDF/dl-PCBs	ndl-PCBs
		(pg WHO-TEQ/g fat)			(ng/g fat)
Free-living cervids (n = 23)	X_avr_ ± SD	2.53 ± 1.42	2.24 ± 1.58	4.77 ± 2.92	5.69 ± 3.57
	range	0.36–5.55	0.43–5.50	0.79–10.80	1.57–14.75
Farmed cervids (n = 11)	X_avr_ ± SD	0.79 ± 0.73	1.06 ± 0.72	1.85 ± 1.21	3.10 ± 1.68
	range	0.23–2.50	0.23–2.80	0.53–3.97	1.38–6.51
Maximum levels (Commission Regulation 2023/915 EU)		3.0	-	7.5	-

1 X_avr_ – average concentration; PCDD/PCDFs – polychlorinated dibenzo-p-dioxins and polychlorinated dibenzofurans; dl – dioxin-like; ndl – non-dioxin-like; WHO-TEQ – World Health Organization toxic equivalency quotient; SD – standard deviation

### Intake of dioxins and dl-PCBs

In the assessment of consumer exposure to dioxins and dl-PCBs against the TWI of 2 pg WHO-TEQ/kg body weight (12) (Table 3), an adult very frequent consumer of farmed game animal meat had an estimated intake of 65% of the TWI. However, in the case of a child very frequent consumer it was 99% of the TWI. The consumption of the more highly contaminated free-living game animal meat (from red deer and roe deer) exposed that adult to dioxins in the amount of 133% of the TWI, and that child to as much as 202% of the TWI of these pollutants.

**Table 3.** Theoretical intake of polychlorinated dibenzo-p-dioxins (PCDDs), polychlorinated dibenzofurans (PCDFs) and dioxin-like polychlorinated biphenyls (dl-PCBs) (expressed as a percentage of the tolerable weekly intake – TWI) by adults and children with muscle tissue from free-living and farmed cervids based on mean and 97.5th percentile (P97.5) values of results

		PCDD/F/dl-PCBs pg WHO-TEQ/g wet weight	Intake (% TWI)					
			Occasionally		Frequent		Very common	
			Adult	Child	Adult	Child	Adult	Child
Free-living cervids	X_avr_	0.34	1.89	2.87	11.0	17.0	84.0	127.0
	P97.5	0.54	2.94	4.62	18.0	27.0	133.0	202.0
Farmed cervids	X_avr_	0.09	0.52	0.78	2.0	3.0	23.0	35.0
	P97.5	0.26	1.45	2.20	9.0	13.0	65.0	99.0

1 X_avr_ – average consumption; WHO-TEQ – World Health Organization toxic equivalency quotient

## Discussion

Wild animals living in a sylvatic environment are constantly exposed to the chemical pollutants present in the abiotic and biotic elements of that environment. The concentration of accumulated substances in the tissues of animals and plants may reach higher levels than in the surrounding environment. The source of dioxins, PCBs and other POPs is also the soil, which wild animals take in with food (1, 14). Exposure to these compounds may be the result of the legacy contamination by dioxins of the environment where animals live as a result of industrial human activity. The extent of animals’ exposure to dioxins may depend on the their migratory habits and the degree of contamination of the areas through which they move. Free-living animals may also contact point sources of contamination (industrial and urban sewage) and surface pollutants washed away with atmospheric precipitation from urbanised, agricultural and forested areas. Pollution from traffic washed off road surfaces and from oil, gas and other pipelines as well as sewage and sediment channels also raises the dioxin burden in a given area. Red and roe deer lead sedentary lifestyles: in favourable conditions they rarely migrate and their living areas are small, ranging from 0.05 to 8 km^2^ (15, 19, 22). Therefore, if exposed to dioxins and PCBs released from local sources, they are constantly exposed. The levels detected in animal tissues may indicate environmental contamination.

Diet is the main source of human exposure to dioxins and PCBs. More than 80% of the daily dose of dioxins and PCBs is absorbed by humans through food of animal origin (5, 8, 10, 27). Fruit, vegetables and water contribute little to overall human exposure to these compounds, being the source of only from 0.01% to 3% (17). In 2012 it was estimated that a significant part of the European population consumes dioxins and dl-PCBs through the diet in the range of 0.57 to 2.54 pg WHO-TEQ/kg b.w. per day (9). These values exceed the safe TWI (10). Research shows that the main sources of dioxins and PCBs are meat and meat products, milk and dairy products and fish (4). However, the percentage of the intake of dioxins and PCBs deriving from a particular food source is variable and depends on the geographical location of a given country and the eating habits of its inhabitants.

The problem of dioxins and PCBs in game meat seems to be so significant that in 2011, the German Federal Institute for Risk Assessment (BfR) issued an opinion on the consumption of game meat, according to which regular consumption of game meat (especially liver) may result in increased intake of dioxins and PCBs, which is harmful to health (3). It is important to keep in mind that in addition to its taste and nutritional value, wild game can also have contamination with POPs. Therefore, it is important to maintain surveillance of this link in the food chain as well.

The observed difference in the accumulation of dioxins and PCBs between the tissues of free-living deer and farmed deer may result from the different characteristics of the two populations’ locations. Deer farms are usually located in ecologically clean areas, while free-living game animals often live adjacent to urbanised areas or industrial districts; therefore, the levels determined in free-living deer tissues are higher.

## Conclusion

The levels determined in the meat of free-living cervids are higher than the levels determined in the meat of farmed counterparts. Meat from free-ranging cervids may be a significant source of dioxins and related compounds for populations that frequently consume game; generally there exists a real health risk to consumers of food originating not only from free-living but also from farmed cervids, albeit a negligible one.
